# A Natural Polymorphism in rDNA Replication Origins Links Origin Activation with Calorie Restriction and Lifespan

**DOI:** 10.1371/journal.pgen.1003329

**Published:** 2013-03-07

**Authors:** Elizabeth X. Kwan, Eric J. Foss, Scott Tsuchiyama, Gina M. Alvino, Leonid Kruglyak, Matt Kaeberlein, M. K. Raghuraman, Bonita J. Brewer, Brian K. Kennedy, Antonio Bedalov

**Affiliations:** 1 Clinical Research Division, Fred Hutchinson Cancer Research Center, Seattle, Washington, United States of America; 2 The Buck Institute for Research on Aging, Novato, California, United States of America; 3 Department of Genome Sciences, University of Washington, Seattle, Washington, United States of America; 4 Howard Hughes Medical Institute, Lewis-Sigler Institute for Integrative Genomics, and Department of Ecology and Evolutionary Biology, Princeton University, Princeton, New Jersey, United States of America; 5 Department of Pathology, University of Washington, Seattle, Washington, United States of America; University of Virginia School of Medicine, United States of America

## Abstract

Aging and longevity are complex traits influenced by genetic and environmental factors. To identify quantitative trait loci (QTLs) that control replicative lifespan, we employed an outbred *Saccharomyces cerevisiae* model, generated by crossing a vineyard and a laboratory strain. The predominant QTL mapped to the rDNA, with the vineyard rDNA conferring a lifespan increase of 41%. The lifespan extension was independent of Sir2 and Fob1, but depended on a polymorphism in the rDNA origin of replication from the vineyard strain that reduced origin activation relative to the laboratory origin. Strains carrying vineyard rDNA origins have increased capacity for replication initiation at weak plasmid and genomic origins, suggesting that inability to complete genome replication presents a major impediment to replicative lifespan. Calorie restriction, a conserved mediator of lifespan extension that is also independent of Sir2 and Fob1, reduces rDNA origin firing in both laboratory and vineyard rDNA. Our results are consistent with the possibility that calorie restriction, similarly to the vineyard rDNA polymorphism, modulates replicative lifespan through control of rDNA origin activation, which in turn affects genome replication dynamics.

## Author Summary

Although many aging regulators have been discovered, we are still uncovering how each contributes to the basic biology underlying cell lifespan and how certain longevity-promoting regimens, such as calorie restriction, manipulate the aging process across species. Since many cellular aging processes between human cells and budding yeast are related, we examined a collection of genetically diverse yeast and discovered that a genetic variant in vineyard yeast confers a 41% lifespan increase. The responsible sequence in the vineyard yeast reduces the amount of DNA replication that initiates at the ribosomal DNA (rDNA) locus, a chromosome-sized region of the genome that is dedicated to the production of ribosomal RNA required for protein synthesis and growth. Strikingly, we find that calorie restriction conditions also reduce rDNA replication, potentially promoting longevity by the same mechanism. While the rDNA has been previously linked to lifespan control, how this single locus affects global cell function has remained elusive. We find that a weakly replicating rDNA promotes DNA replication across the rest of the cell's genome, perhaps through the re-allocation of replication resources from decreased rDNA demand. Our findings suggest that the cell's inability to complete genome replication is one of the major impediments to yeast longevity.

## Introduction

The budding yeast *Saccharomyces cerevisiae* has become a favorite model for studying the genetic and molecular basis for variation in lifespan [1]. In particular, the unequal division of mother and daughter cells in this species makes it especially amenable for analysis of replicative lifespan (RLS), the number of “daughter” cells that a “mother” cell can produce before it senesces, which is typically 20–30 [2]. RLS is thought to be analogous to aging of mitotic cells such as stem cells and epithelia. Genetic screens for RLS alteration have identified many genes whose deletion confers lifespan extension, such as those in growth and metabolism pathways, and even more genes whose absence reduces yeast longevity [3].

The discovery that long-lived mutants redirect the SIR (Silent Information Regulator) complex, including the NAD-dependent histone deacetylase Sir2, to the nucleolus first implicated events at the rDNA as potential determinant of aging [4]. The identification of the rDNA binding protein Fob1 as a regulator of yeast lifespan further supported rDNA's involvement in aging [5]. The rDNA locus is a highly repetitive region of the genome, over 1MB in size, consisting of approximately 150 tandem repeats of a 9.1 kb sequence that encode the 35S transcription unit (18S, 5.8S, 25S RNAs transcribed by RNA polymerase I) and the 5S gene (transcribed by RNA polymerase III). More than 60% of all transcription in a yeast cell is dedicated to the production of these rRNAs [6], which can be accomplished only through the concurrent transcription from multiple rDNA repeats [7]. Each repeat also contains non-transcribed regions with features that allow it to maintain its chromosome-like size, such as origins of replication. An RNA polymerase II promoter (E-pro) is also found in one of the ‘non-transcribed” regions. It is this promoter whose activity is suppressed by Sir2. In the absence of Sir2, RNA polymerase II transcription disrupts rDNA-bound cohesin [8] leading to an increase in intra-chromatid mitotic recombination and the formation of extrachromosomal rDNA circles (ERCs) [9]. ERCs have been shown to preferentially segregate to mother cells and their accumulation in old mother cells has been proposed as a cause of aging in yeast, perhaps through sequestration of as yet unknown detrimental factors [10]. An attractive feature of the ERC model is that it could explain how daughters can be rejuvenated, i.e. how daughters can have the same RLS regardless of whether they came from old or young mothers. Accumulation of ERCs in old mothers has been proposed to be responsible for the decreased lifespan seen in *sir2Δ* mutants [11] and, conversely, the increased lifespan of strains that contain an extra genomic copy of *SIR2*. The Fob1 protein binds to the rDNA replication fork barrier (RFB), creating a unidirectional replication block thought to prevent the head-on collision between the rRNA Polymerase I transcription machinery and the DNA replication machinery [12], [13]. However, Fob1 binding also promotes rDNA recombination by increasing the probability of double strand breaks and hence promoting ERC formation. As expected for a gene whose deletion decreases ERC accumulation, *fob1Δ* mutants have increased RLS.

Calorie restriction is the best-studied intervention that promotes longevity [14]. Restriction of dietary intake to 70% *ad libitum* was first reported to extend the lifespans of mice and rats [15], [16], [17]. Since then, calorie restriction-mediated lifespan extension has also been documented in yeast, worms, flies, and several other organisms [14], [18], [19]. Significant progress has been achieved in dissecting the nutrient responsive signaling pathways responsible for this lifespan extension, some of which, like the TOR pathway, are shared between species as distant as mice and yeast. The downstream effectors of these signaling pathways, on the other hand, are unknown. It is known, however, that Sir2 and Fob1 act in a pathway that is genetically distinct from calorie restriction and that calorie restriction is able to extend the lifespan of *sir2Δ fob1Δ* double mutants [20], [21]. The mechanism through which calorie extension extends lifespan is therefore not via direct regulation of Sir2 or Fob1 and remains mysterious.

To begin to address these gaps in our knowledge we used an outbred yeast model, consisting of strains generated in a cross between the S288c laboratory strain BY4716 (BY) and the vineyard strain RM11-1a (RM), to identify naturally occurring polymorphisms that regulate RLS and found that the rDNA locus is the major regulator of lifespan in this cross. Among the rDNA sequences that differ between RM and BY, we identified a polymorphism in the RM rDNA locus that leads to a marked reduction in rDNA origin activity both in a plasmid assay and at its native location in the genome. The less active RM rDNA origin confers a reduced size of the rDNA array and extends lifespan. Critically, this lifespan extension is independent of both Sir2 and Fob1 but the activity of both of these rDNA origins is strikingly affected by calorie restriction, thus suggesting a replication-based mechanism for calorie restriction-mediated extension of RLS.

## Results

### Replicative lifespan analysis of an outbred segregant library

**Figure 1:**
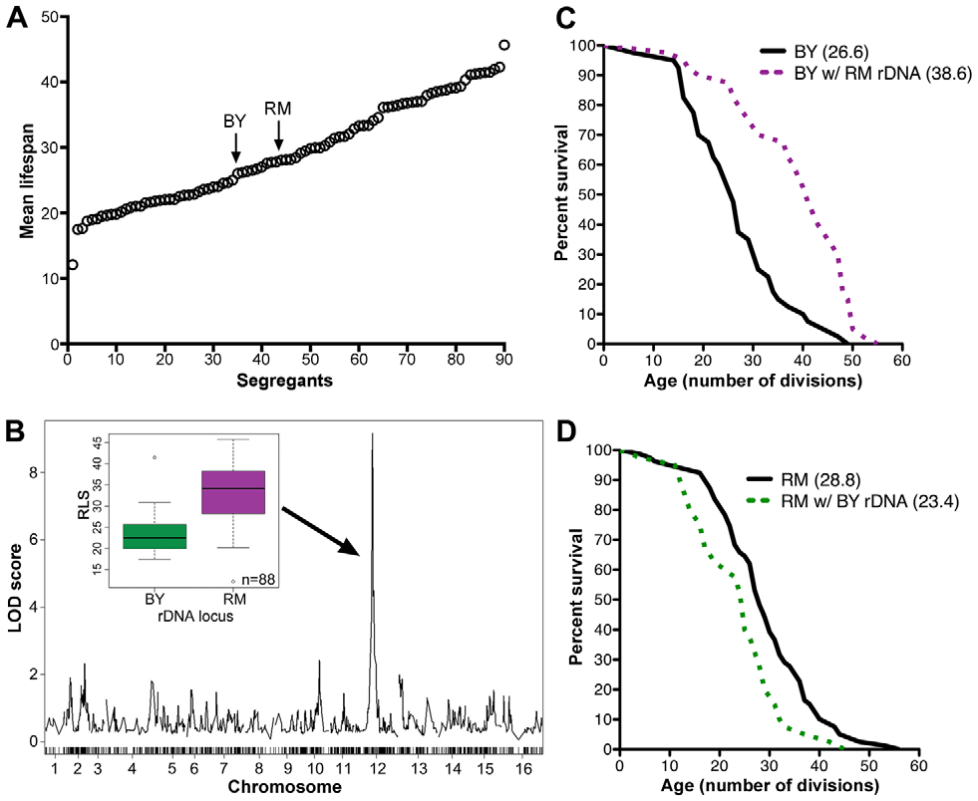
rDNA is the major regulator of replicative lifespan in the RM/BY cross. (A) Replicative lifespans (RLS) of the RM and BY parental strains and 88 segregant strains were determined by mother-daughter separation on YPD plates. The mean RLSs were determined by examining 20 cells of each strain. (B) A genome-wide scan to find loci that contribute to lifespan reveals a maximum LOD score centered on the rDNA locus on chromosome XII. RLS distributions of strains inheriting the BY or RM rDNA loci are shown in the inset. Average RLS for strains with BY rDNA was 23.5±4.6 and average RLS for strains with RM rDNA was of 33.1±7.0 (*P* = 9.5×10^−12^, ν = 88). (C) RLS curves (n = 40 cells) for the BY parent and the backcrossed BY strain with RM rDNA (*P* = 10^−3^, ν = 2). (D) RLS curves (n = 40 cells) for the RM parent and backcrossed RM strain with BY rDNA (*P* = 5.0×10^−3^, ν = 2). Mean RLS for backcross strains are indicated in parentheses (C, D).

We measured the RLS of 20 individual cells from each of 88 meiotic segregants previously derived from a cross between a laboratory yeast strain BY4716 (BY parent) and a vineyard yeast strain RM11-1a (RM parent) [22]. We found continuous variation in lifespan between the segregants, with mean RLS ranging from 12 to more than 40 generations (Figure 1A), suggesting that multiple loci are involved in controlling longevity. This wide variation of RLS among the segregants is derived from parents with similar lifespans (26.5 and 28.6 generations for BY and RM respectively; Figure 1A). Such transgressive segregation, in which the progeny have more extreme phenotypes than the parents, suggests the presence of multiple loci that have compensatory effects in the parental strains. To determine the extent to which the observed lifespan variation was genetically determined, we repeated lifespan analysis on 40 of the segregant strains (data not shown). Heritability was 82%, indicating that a large fraction of lifespan variation in this cross is determined genetically.

Genome-wide linkage analysis revealed strong linkage to a locus on chromosome XII (LOD score of 9; Figure 1B, Table S2). Further investigation refined this linkage to the 1.2 Mbp region corresponding to the rDNA locus. Only one crossover event between the markers flanking the rDNA region (potentially a mitotic crossover in the diploid) was found within segregants, consistent with the previous observation that rDNA is inherited as a single Mendelian locus [23]. We determined that 46% of heritable lifespan variation (38% of total variation) between the segregants is controlled by the rDNA locus, indicating that rDNA is the major regulator of lifespan in this cross. The segregants that inherited the rDNA locus from the BY parent had an average RLS of 23.5±4.6 (Figure 1B inset), comparable to lifespans previously seen from laboratory strains such as s288c and W303. In contrast, segregants that inherited this locus from the RM parent had an average RLS of 33.1±7.0 cell divisions (Figure 1B inset), a 41% increase in longevity (*P* = 9.5×10^−12^, ν = 88).

To isolate the effects of the rDNA locus from other genomic polymorphisms, we carried out eight sequential backcrosses to the BY laboratory strain while maintaining the RM vineyard rDNA. This backcrossed strain had a mean lifespan of 38.6, a 45% increase compared to the mean of 26.6 for the BY parental strain (*P* = 10^−3^, ν = 2, Figure 1C). Conversely, we used eight backcrosses to transfer the laboratory rDNA into the RM background. The RLS of the RM parental strain at 28.8 was 23% longer than to the RLS of 23.4 in the outcrossed RM strain containing the laboratory rDNA, (*P* = 5.0×10^−3^, ν = 2, Figure 1D). Thus, consistent with mapping results, the backcross analysis in both parental backgrounds confirmed that the rDNA has a dramatic effect on lifespan.

### rDNA affects lifespan independently of Sir2, Fob1, and ERCs

**Figure 2:**
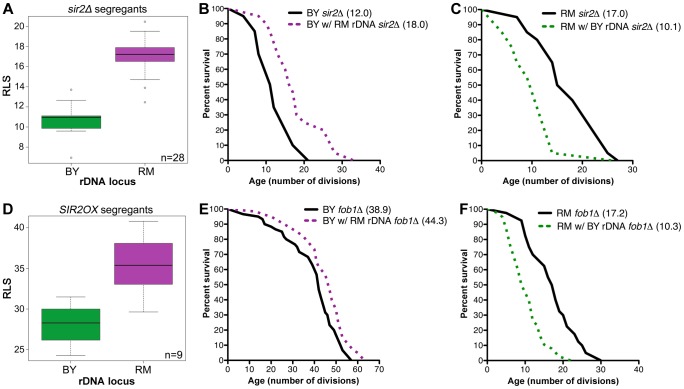
rDNA inheritance regulates lifespan independently of Sir2 and Fob1. (A and D) Mean RLS distribution of 28 segregants with *SIR2* deleted (A) or 9 segregants overexpressing *SIR2* (D). Segregants with the RM rDNA locus were longer-lived than those inheriting the BY rDNA locus even in the absence of *SIR2* (17.0±2.1 vs. 10.8±1.7, *P* = 6.1×10^−9^, ν = 2) or with *SIR2* overexpression (35.4±3.4 vs. 28.1±3.0, *P* = 2.0×10^−2^, ν = 2). (B, C, E and F) Lifespan measurements (n = 40 cells) for the backcrossed BY and RM strains containing BY or RM rDNA and *sir2Δ* or *fob1Δ.* Mean lifespans for each strain are given in parentheses.

To investigate whether the lifespan effect of the rDNA is mediated by Sir2, we deleted *SIR2* in 28 randomly selected segregant strains and measured their replicative lifespans. While the average lifespan of all strains dramatically decreased upon *SIR2* deletion, *sir2Δ* segregant strains with RM rDNA had significantly longer RLS (17.0±2.1) compared to their BY rDNA counterparts (10.8±1.7, *P* = 6.1×10^−9^, ν = 2) (Figure 2A). Sir2 function is therefore not required for RM rDNA-induced increase in RLS. The Sir2-independence of the rDNA's effect on lifespan was recapitulated in our backcrossed strains: BY *sir2Δ* strains with RM rDNA fared better than the parental strain, with lifespan means of 18.0 and 12.0, respectively (*P* = 1.7×10^−3^, ν = 2, Figure 2B). The RM *sir2Δ* strain also lived longer (17.0) than the BY rDNA *sir2Δ* strain (10.1, Figure 2C, *P* = 9.0×10^−4^, ν = 2). Additionally, we generated segregant strains in which an extra copy of *SIR2* was inserted in the genome and found that the longevity benefit of the RM rDNA locus was still maintained in the presence of *SIR2* overexpression (35.4±3.4 vs. 28.1±3.0 divisions, Figure 2D, *P* = 2.0×10^−2^, ν = 9). These results demonstrate that the effect of the rDNA locus on lifespan extension is independent of Sir2.

To determine whether Fob1 is required for the rDNA's effect on lifespan, we deleted *FOB1* both in the parental strains and in the backcrossed strains. In the BY background, *FOB1* deletion led to increased lifespan in both the strain with BY rDNA (38.9) and the strain with RM rDNA (44.3, *P* = 6.7×10^−3^, ν = 2, Figure 2E). Since the RM rDNA locus still conferred lifespan extension in the absence of Fob1, we conclude that the longevity benefit of the RM rDNA is not dependent on the presence of Fob1. Unexpectedly, in the RM background, loss of Fob1 reduced lifespan by half, an effect that occurred in both the RM parental strain (17.2) and the strain with BY rDNA (10.3, Figure 2F). Thus it appears that in the RM strain, unlike most of the laboratory strains, *FOB1* deletion decreases lifespan through unknown mechanisms. Nonetheless, the longevity benefit of the RM rDNA locus persisted (*P* = 10^−4^, ν = 2). Our results thus demonstrate that the effect of the rDNA on life span is independent of Fob1.

**Figure 3:**
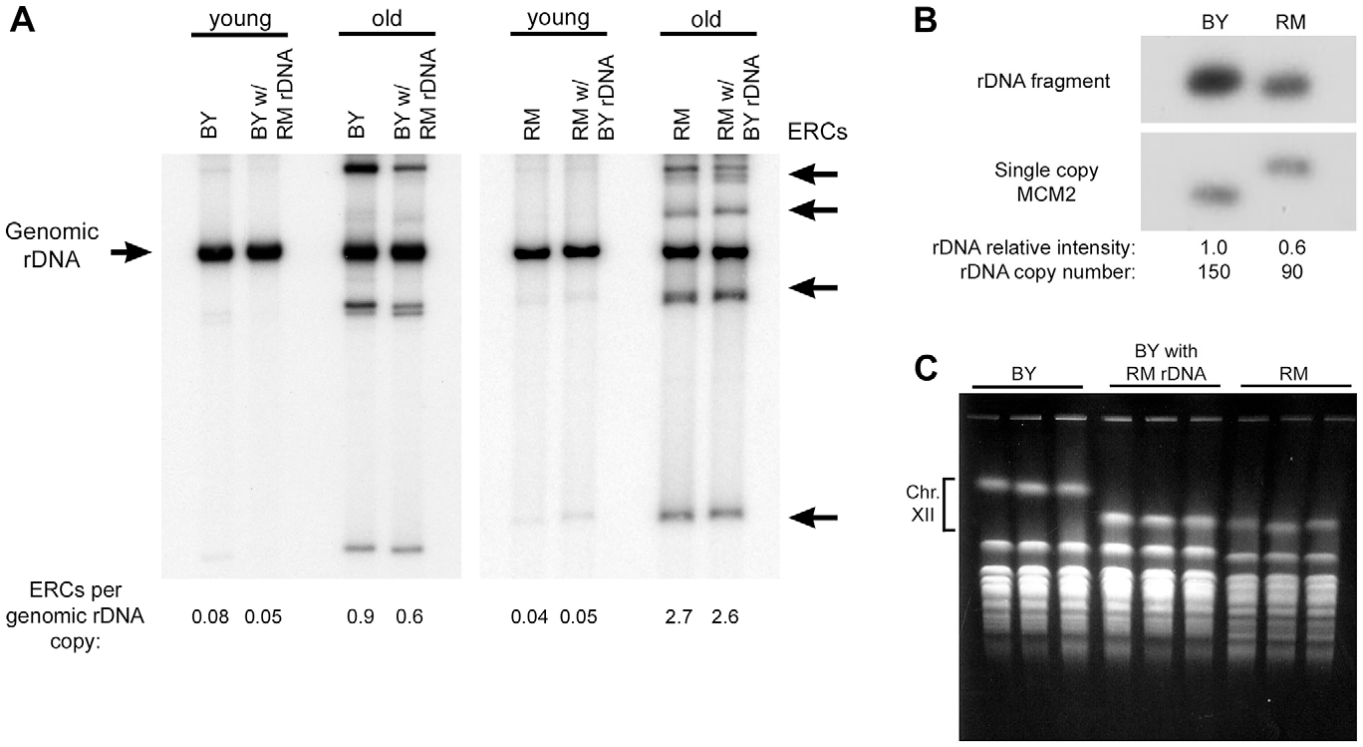
ERC abundance and rDNA copy number. (A) The relative abundance of ERCs in the backcrossed RM and BY strains with either BY or RM rDNA was determined in populations of old and young cells. DNA (uncut) was separated on a 0.8% agarose gel and a Southern blot was probed for rDNA. Chromosomal rDNA (Genomic rDNA) runs with limiting mobility; ERCs of varying repeat numbers and topological properties generate multiple distinct bands. (B) To measure relative differences in rDNA copy number, genomic DNA from BY and RM strains was digested with BglII and a Southern blot was sequentially probed for the single copy *MCM2* gene and an rDNA fragment. (There is a restriction polymorphism near *MCM2* that distinguishes the two strains.) Assuming BY has 150 rDNA repeats, RM has ∼90. (C) Three independent clones from the BY strain, the RM strain and the BY strain with the RM rDNA were examined by CHEF gel electrophoresis to determine the size of chromosome XII and thereby confirm the size difference of the rDNA locus. A photograph of the ethidium bromide stained gel is shown.

Intrachromosomal recombination between the tandem rDNA repeats generates extrachromosomal rDNA circles (ERCs), whose preferential accumulation in mother cells has been proposed as one of the mechanisms that limits replicative potential [10], [24]. To determine if vineyard and laboratory rDNA loci differ in their ability to generate and/or maintain ERCs, we measured ERC levels in young logarithmically growing cells and in a purified population of old mother cells of both parental strains and their respective backcrossed rDNA replacement strains (Figure 3A). As expected, we observed an increase in ERCs in old cells compared to young cells in both parental backgrounds. In the BY background, old cells with RM rDNA had 33% fewer ERCs compared to cells with the parental BY rDNA. However, in the RM background, the amount of circles was comparable between the strains with RM rDNA and the strain with BY rDNA, demonstrating that the longevity benefit of the RM rDNA cannot be attributed to different propensities of the two rDNAs to accumulate circles in old mothers. Together, these results suggest that the effect of the rDNA on lifespan occurs though a mechanism that is independent of Sir2, Fob1, and ERC accumulation.

To determine if BY and RM rDNA differ in their capacity to transcribe rRNA and generate ribosomes, and thus to affect global protein translation and growth, we measured doubling time and cell size in logarithmically growing BY strains with RM and BY rDNA. We found that both doubling time and cell size are essentially indistinguishable in the two strains (Figure S1), indicating that the capacity for new biomass generation, the best measure of global protein synthesis [6], [25], is equal for the two rDNA arrays, from which we infer that ribosome biogenesis and rDNA transcription are also equal.

### BY and RM rDNAs differ in copy number and intergenic sequence polymorphisms

To determine if the number of the rDNA repeats differed between the two parental strains, we performed quantitative Southern blot analysis (Figure 3B). The number of rDNA repeats in the vineyard strain was approximately 60% of that in the laboratory strain (∼90 vs. ∼150 copies [26], respectively). We confirmed that RM rDNA is shorter than BY rDNA using contour-clamped homogeneous electric field (CHEF) gel electrophoresis. Remarkably, the reduced size of the vineyard rDNA locus, estimated from the size of chromosome XII by CHEF gel electrophoresis (Figure 3C), was preserved after 10 successive backcrosses to the laboratory strain (∼500 population doublings). Conversely, the back crossed strain with the BY rDNA maintained its larger rDNA (data not shown). The copy number of the rDNA array is therefore determined by cis-acting sequences within the repeats themselves.

**Figure 4:**
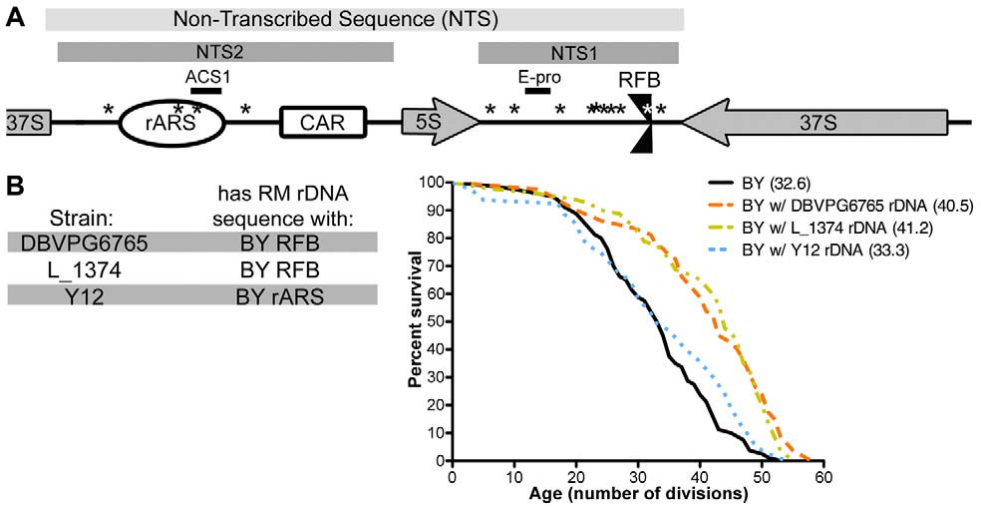
Polymorphism in the rDNA origin of replication mediates replicative lifespan. (A) A schematic of rDNA elements and identified polymorphisms, not drawn to scale. Locations of sequence differences between the RM and BY rDNA are noted with asterisks (*). (B) Wild yeast isolates DBVPG6765, L_1374, and Y12 have RM rDNA sequences with the single BY RFB or BY rARS polymorphisms. rDNA from each of these wild strains was backcrossed into the BY background and lifespan analysis was performed on each (n = 40 cells). The mean replicative lifespan for each strain is indicated in parentheses. rDNA loci from DBVPG6765 and L_1374 confer significant lifespan extension (*P* = 10^−4^, ν = 2) while the Y12 rDNA locus does not (*P* = 1.4×10^−1^, ν = 2).

Since our results suggest that the rDNA sequence itself is important for determining longevity as well as rDNA copy number, we compared the sequences of RM and BY rDNAs. We found no sequence polymorphisms in the 37S and 5S regions encoding rRNA transcripts, consistent with the high degree of conservation of these regions. However, we found that the non-transcribed spacer (NTS) regions are highly divergent between the two strains (Figure 4A). Within the NTS are several elements that have been found to play an important role in the maintenance of the rDNA locus: NTS2 contains the rDNA origin of replication (rARS or rDNA autonomously replicating sequence) [27] and the cohesin associating region (CAR) [28]; NTS1 contains a binding site for Fob1 that creates a unidirectional replication fork barrier (RFB) [12] and the E-pro RNA Polymerase II promoter which is silenced by Sir2 [8]. We identified sequence changes in the rDNA origin of replication (rARS) and in the replication fork barrier (RFB). We focused our attention on these two polymorphisms because they alter known functional elements. Using high-throughput sequencing, we found that the identified RFB and rARS variants are homogenous within a strain, with at most one RM repeat in the BY rDNA and vice versa. The homogeneity within the rDNA array allows us to investigate the effects of an rDNA array as a single sequence rather than a complex mixture of heterogeneous repeats.

To assess how the rARS and RFB polymorphisms affect lifespan, we examined rDNA sequences from a diverse collection of wild yeast. The majority of the 37 *S. cerevisiae* isolates sequenced by the Saccharomyces Genome Resequencing Project (SGRP) [29], [30] possessed the RM rDNA sequence, with only a handful having those polymorphisms seen in the BY strain. Fortuitously, we found several strains that have “hybrid rDNA” sequences: strains whose rDNA contains the BY rARS and the RM RFB or vice versa. To distinguish the effects of the rARS alterations from those at the RFB, we generated outcrossed strains in the BY background with rDNA from three *S. cerevisiae* isolates with “hybrid rDNA” sequences. The rDNA sequences from the strains DBVPG6765 and L_1374 have the RM ARS and the BY RFB. Backcrossed strains with either of these rDNA sequences had significant lifespan extension (*P* = 10^−4^, ν = 2) on par with that of RM rDNA (Figure 4B), suggesting that the RM rARS polymorphism rather than the RM RFB polymorphism confers increased lifespan. In contrast, when we examined rDNA from the sake strain Y12, which has the BY rARS and the RM RFB, we found no extension of lifespan (*P* = 0.1404, ν = 2). Therefore, we conclude that the RM rARS polymorphism, not the RM RFB polymorphism, is responsible for conferring lifespan extension.

### RM rDNA ARS polymorphism reduces ARS activity

**Figure 5:**
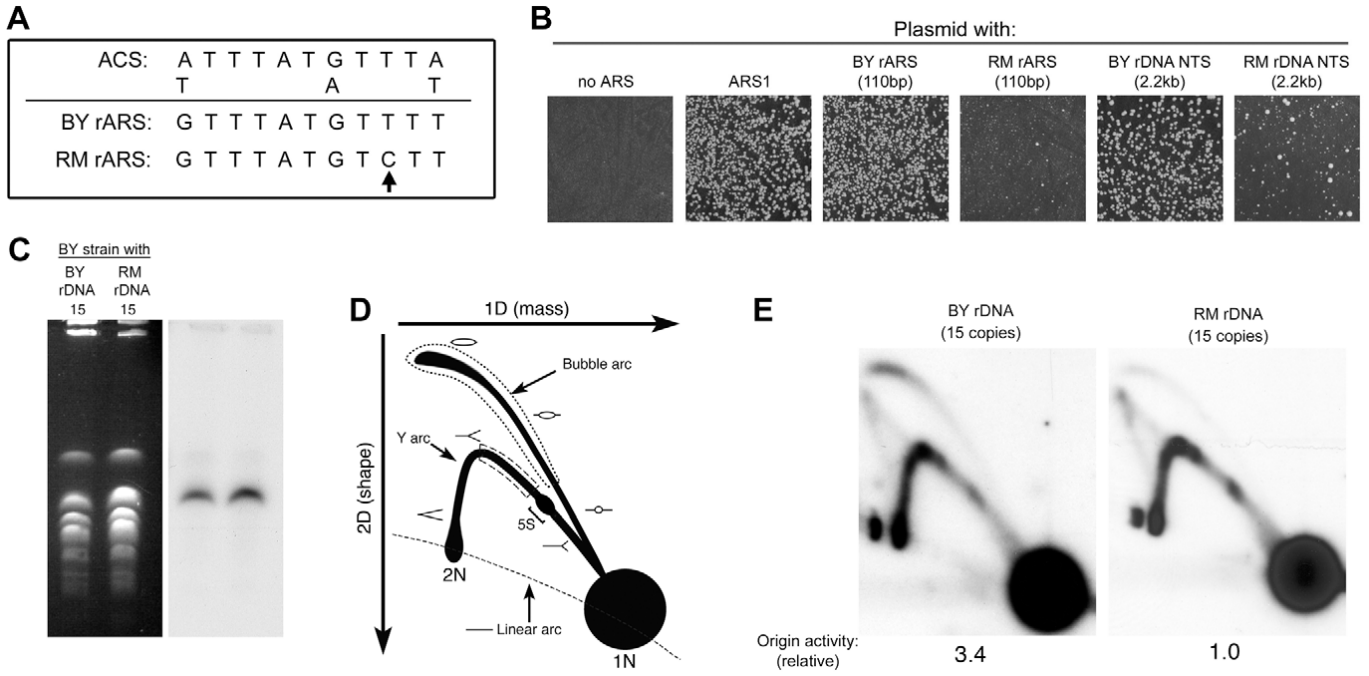
Difference in rDNA origin activity in the RM and BY rDNA loci. (A) Sequence comparison of ACS1 in the BY and RM rARSs. The sequence polymorphism in the RM rARS ACS1 is indicated by an arrow. (B) Replication origin sequences from a variety of sources were cloned into a vector containing the KanMX6 gene. The recombinant plasmids were then transformed into the BY strain. After a day of growth on YPD medium, the transformants were replica-plated onto YPD plates with G418 to select for cells with the recombinant plasmid. A plasmid with no ARS and a plasmid with the strong chromosomal origin *ARS1* serve as the negative and positive controls, respectively. Plasmids containing the rARS from the BY rDNA locus, either as a 110 bp fragment or a 2.2 kb fragment encompassing the entire NTS region, transformed the BY strain at high frequency producing >1000 transformants/microgram of plasmid DNA. Plasmids containing the rARS from the RM rDNA locus, either as a 110 bp or a 2.2 kb fragment encompassing the entire NTS region, transformed much more poorly with <200 transformants per transformation. The colony sizes of these transformants were also more variable. (C) Chromosomes separated on a CHEF gel stained with ethidium bromide (left) or blotted and probed for rDNA (right) verify that chromosome XII is approximately the same size in the engineered RM and BY strains. The size of chromosome XII relative to other chromosomes of known size on the CHEF gel suggest that both strains have rDNA loci that contain ∼15 copies of the rDNA repeat. (D) Schematic diagram illustrating the replication intermediates that can be resolved by two-dimensional (2D) gel electrophoresis. To estimate relative origin efficiencies, the abundance of intermediates along the bubble arc is compared to the intermediates in the ascending arm of the Y arc (dotted and dashed regions, respectively). A bracket denotes the positions of paused replication forks at the 5S rRNA gene. (E) The 4.7 kb NheI fragments containing the rDNA ARS from a BY strain with ∼15 copies of either BY or RM rDNA repeats (in a *fob1Δ* background) are examined by Southern blotting of a 2D gel. The ratio of bubble∶Y was defined as 1.0 for the RM rDNA strain. In comparison, the normalized bubble∶Y ratio for the BY strain is 3.4.

Since the rate of rDNA amplification has been found to correlate with rDNA origin activity [31], we suspected that the reduced array size of the RM rDNA compared to the BY rDNA could be a consequence of reduced origin activity conferred by the RM rARS polymorphism. The RM rDNA origin of replication has a cytosine polymorphism at a highly conserved thymine residue in the A/T-rich rDNA ACS1 sequence (Figure 5A), suggesting that it may impair origin function and DNA replication [27], [32], [33]. To compare origin activity of the different rDNA sequences, we cloned the rARS sequences from RM and BY rDNA into an origin-free vector containing a KanMX marker and tested the ability of these sequences to promote autonomous plasmid maintenance upon transformation into a BY host strain. We find high-frequency transformation with the BY rARS confirming robust ARS function; the RM rARS transforms at a greatly reduced frequency and the colonies that arise are variable in size (Figure 5B). Use of the entire 2.2 kb rDNA NTSs likewise resulted in greatly disparate ARS activity, eliminating the possibility that different sites within the NTS were serving as origins in the two rDNAs. An origin-free plasmid (no ARS) and a plasmid with a highly active yeast origin (*ARS1*) served as negative and positive controls, respectively. Thus the RM rARS, which is the most common among wild *S. cerevisiae*, and which confers lifespan extension, is much weaker than the BY ARS.

The extreme differences in ARS activity from our plasmid maintenance assay suggest that the replication of the RM rDNA locus could be very different from that of BY. Since the size of the rDNA has been previously shown to affect origin activity [34], presumably by affecting the fraction of actively transcribed repeats, we deemed it important to compare origin activity between strains containing BY and RM rDNA that have the same number of repeats, and therefore, the same number of actively transcribed repeats. As French et al. [7] observed that in strains with fewer than 30 repeats all of the rDNA subunits were actively transcribed, we used a method described previously by Kobayashi *et al*. [35] to generated BY strains containing 15 copies of either the RM or the BY rDNA (Figure 5C). To examine origin activity at the endogenous rDNA locus, we used 2D gel electrophoresis to compare the fraction of repeats with an active origin (bubble-shaped intermediates) to the fraction of repeats that are passively-replicated (Y-shaped intermediates) (Figure 5D). The relative origin activity can then be estimated as the ratio of bubble arc intensity to that of the Y arc. We find that the origin activity of the RM rDNA origin is strikingly reduced (∼3-fold) compared to that of the BY rDNA origin (Figure 5E), consistent with our results from plasmid transformation assays.

### rDNA origin activation modulates genome-wide DNA replication

**Figure 6:**
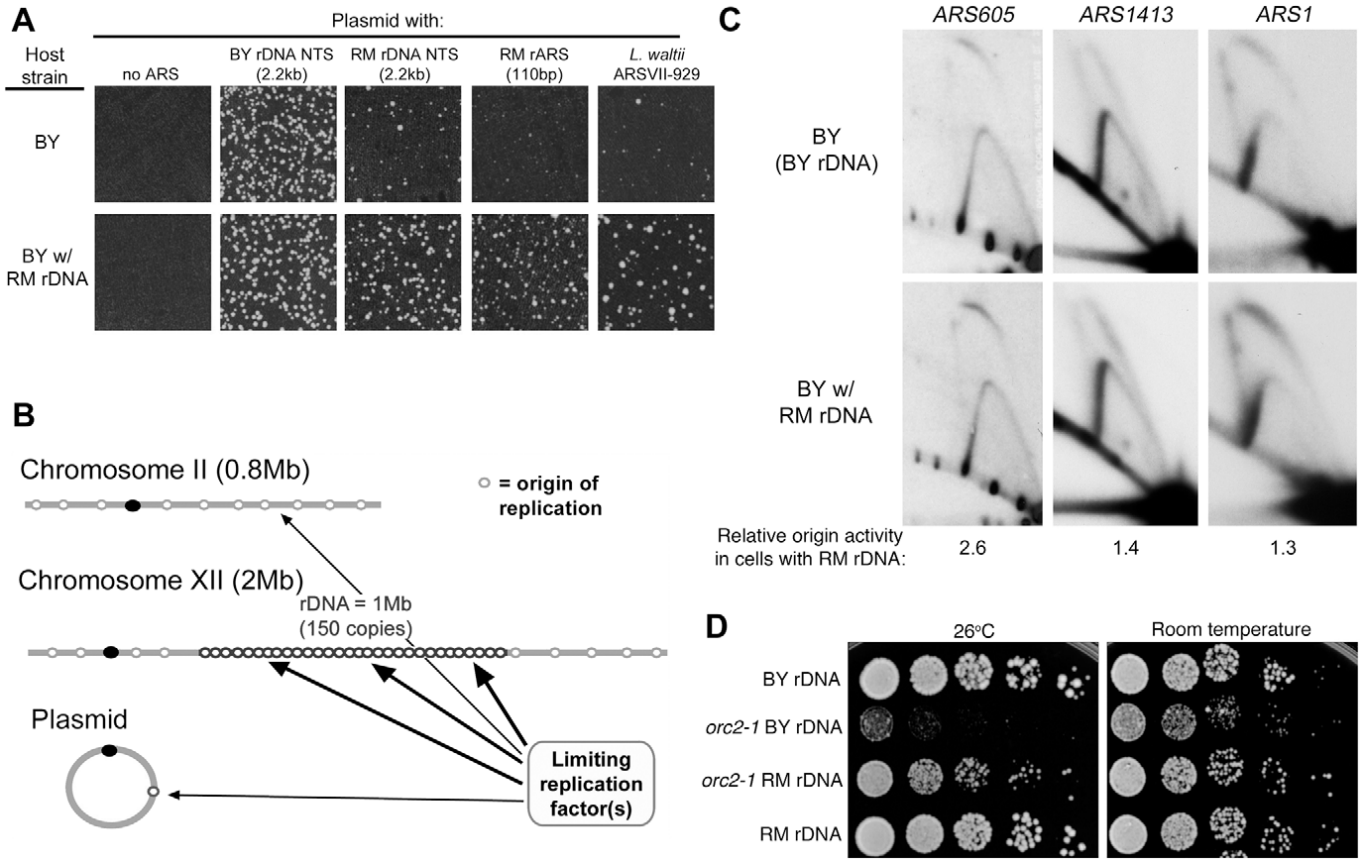
rDNA and genome replication. (A) Replication origins cloned into the KanMX6 vector were transformed into the BY strain and the BY strain with the RM rDNA as described in Figure 5. Transformation efficiency of the BY rDNA ARS was comparable in the two strains. Weak ARSs that produce few transformants in the BY background (2.2 kb RM NTS, 110 bp RM rARS, and *L. waltii* ARSVII-929) show a 5 to 10-fold increase in the frequency of transformation when the BY strain with the RM rDNA is used as a host. (B) A proposed model to explain the effect of the source of the rDNA locus on high-frequency ARS transformation and chromosomal origin firing. The rDNA locus competes with plasmid and genomic origins of replication (for example, origins on chromosome II) for an unknown limiting replication factor. (C) 2D gel analysis of genomic origins *ARS605*, *ARS1413*, and *ARS1* in the BY strain with either BY rDNA or RM rDNA. Enzyme digests, fragment sizes and probes for these three origins can be found in Experimental Procedures. Relative origin efficiencies (bubble∶Y ratios) are normalized to the efficiency observed in the BY strain with BY rDNA. (D) Five-fold serial dilutions of isogenic BY *orc2-1* temperature sensitive strains with either BY or RM rDNA grown at room temperature and 26°C, the semi-permissive temperature for *orc2-1*. RM rDNA locus improves viability of the *orc2-1* mutant.

In the course of our studies of RM rDNA origin usage, we noticed that while the RM rARS performed poorly in the plasmid maintenance assay described above when the BY parent was used as a host, the RM rARS plasmid performed well when the RM parent was used as a host (Figure S2). We then surmised that there is a host factor that is critical for determining the efficiency of a plasmid DNA replication origin. Further investigation revealed that the host factor responsible was the endogenous rDNA array: the weak RM rDNA rARS performed poorly in the plasmid transformation assay when the backcrossed RM strain with the BY rDNA array was used (Figure S2) but performed well when the backcrossed BY strain with the RM rDNA was used (Figure 6A). To determine whether this effect on replication, like the effect on longevity, is specific to the replication origin polymorphism in the host rDNA array, we performed plasmid transformation assays in our backcrossed strains with “hybrid rDNA” sequences containing either the RM rARS polymorphism with the BY RFB polymorphism or vice versa. The backcrossed BY strain with rDNA from DBVPG6765, which contains the RM replication origin and the BY RFB, were transformed at high efficiency by plasmids with RM NTS and RM rARS sequences (Figure S2). In contrast, a backcrossed BY strain with Y12 rDNA, which contains the BY replication origin and the RM RFB, performed poorly in this assay. To address the possibility that our observations were an artifact of the plasmid containing an rDNA sequence, which could possibly direct them to the nucleolus where they would be affected by the same replication forces governing the endogenous rDNA, we tested a plasmid containing a weak non-rDNA origin sequence from *Lachancea waltii* which previously has been shown to have ARS function in *S. cerevisiae* [36]. Consistent with our results with rDNA origins, the *L. waltii* plasmid was more effective in transforming the BY strain that contained the RM rDNA than the complementary strain, demonstrating that these plasmid transformation effects are not specific to plasmids containing rDNA sequences (Figure 6A). We conclude that the replication origins in the rDNA array exert a powerful influence on the ability of weak replication origins, whether from the rDNA or elsewhere, to support plasmid replication.

### RM rDNA locus confers increased activity on weak genomic origins and suppresses effects of the *orc2-1* mutation

Why would having an impaired rARS in the rDNA array lead to greater plasmid origin activity and extended lifespan? We hypothesize that having fewer active rDNA origins would reduce competition for replication factors and therefore increase the potential for replication of plasmids with weak origins (Figure 6B). It is important to note that there are ∼300 origins of replication in the yeast genome outside of the rDNA and, in strains with the BY rDNA locus, and an additional 150 rDNA origins. Origins in the rDNA locus can thus comprise one third of the total genomic origins. If the RM rDNA sequence promotes usage of weak origins on a plasmid through increased availability of replication factors, we surmised that the presence of the RM rDNA might also promote origin activity across the genome. We used 2D gel electrophoresis to examine origin activity at two strong origins (*ARS1* and *ARS1413*; origin efficiencies of 94% (B.B., unpublished data) and 85% [37], respectively) and one weak origin (*ARS605*; origin efficiency 27% [38]) in strains with either RM or BY rDNA. Consistent with our model, we found that activity of the inefficient *ARS605* origin was increased by the presence of the RM rDNA locus (Figure 6C), while the two strong origins were unaffected. The *ARS605* origin was previously noted to have different efficiencies between two different strains, A364A [38] and DKD-5D-HD [39], possibly reflecting rDNA sequence differences. Our results suggest that the RM rDNA locus increases efficiency at a subset of origins, namely inefficient origins, both on plasmids and throughout the genome.

Our results lead us to the hypothesis that the weaker RM rDNA origin is globally compensating for a naturally limiting, unknown replication factor, allowing non-rDNA origins greater access to this limiting factor and promoting replication of the rest of the genome. If this hypothesis is correct, then under conditions where genome replication is specifically compromised by a temperature sensitive mutation in one of the components of replication initiation, we would expect to see the presence of the RM rDNA locus to suppress, at least partially, the growth defect of the temperature sensitive mutation. We created BY strains (with either the RM or BY rDNA) in which replication initiation potential would be limited by the presence of the temperature-sensitive *orc2-1 mutation* [40], [41]. Orc2 is a subunit of the origin recognition complex (ORC) that recruits replication factors to *S. cerevisiae* origin sequences, and the *orc2-1* mutant allele generates an ORC that is unstable at high temperature. An *orc2-1* mutant with BY rDNA is unable to grow at the non-permissive temperature of 26°C and also exhibits slower growth at room temperature. An *orc2-1* mutant with RM rDNA grows as well as an *ORC2* strain at room temperature and significantly improves viability at 26°C (Figure 6D). These results are consistent with the hypothesis that the more efficient rDNA origins in the BY rDNA array can stress global replication. We conclude that having weaker and fewer origins at the rDNA locus can facilitate genome-wide replication.

### Calorie restriction reduces rDNA origin activity

**Figure 7:**
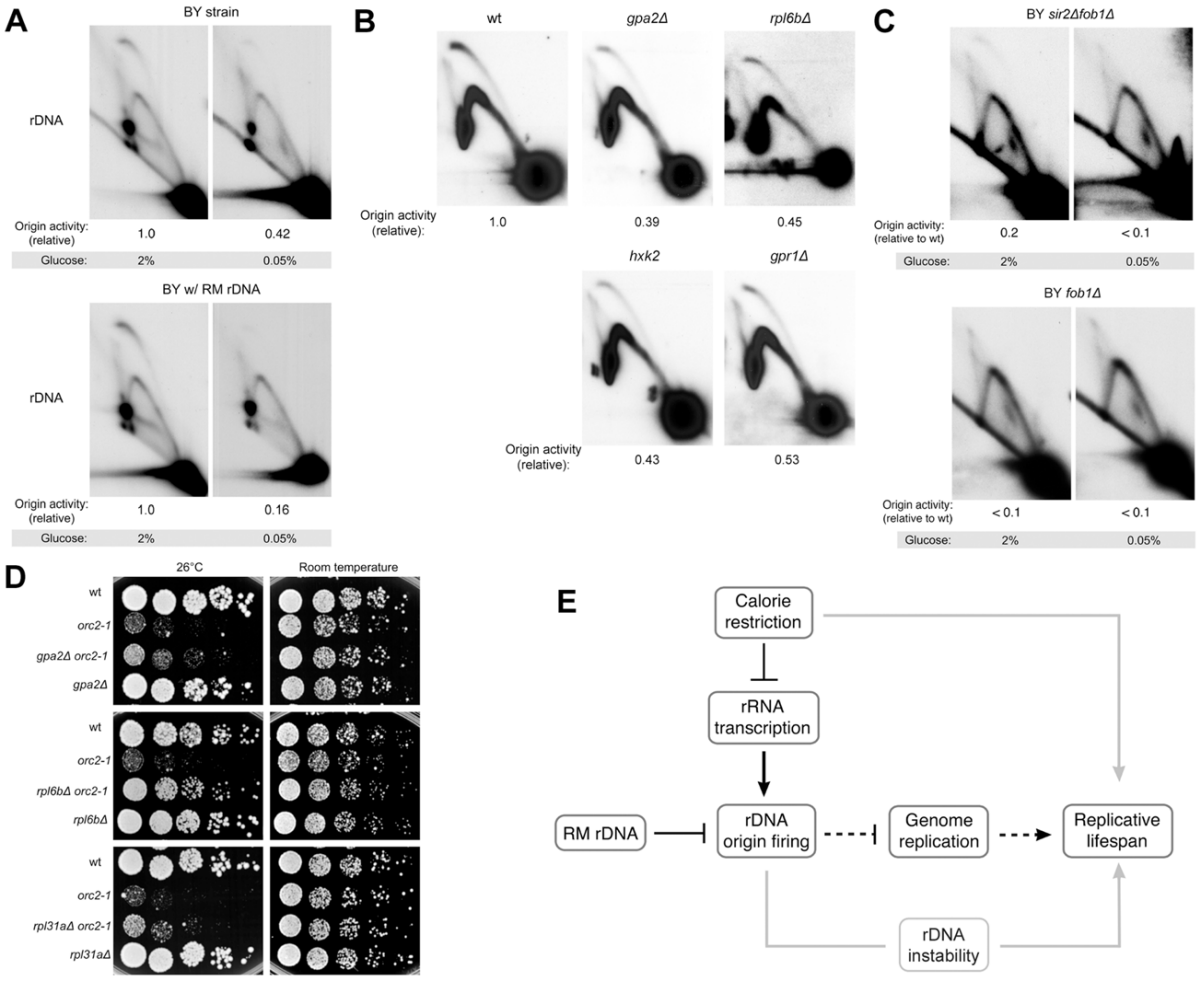
rDNA origin activity is reduced by caloric restriction. (A–C) 2D gel analysis of replication intermediates of the 4.7 kb NheI rDNA ARS fragments. (A) BY strains containing either RM or BY rDNA grown in high (2%) glucose medium or calorie-restricted (0.05%) medium. (B) BY strains with BY rDNA in conjunction with each of the calorie restriction-mimetic mutations *gpa2Δ*, *rpl6bΔ*, *hxk2Δ* and *gpr1Δ* grown in 2% glucose medium. (C) BY strains with either *fob1Δ* or *fob1Δ sir2Δ* grown in high (2%) glucose medium or calorie-restricted (0.05%) medium. (D) Five-fold serial dilutions of isogenic BY strains grown at room temperature and 26°C, the semi-permissive temperature for *orc2-1*. Calorie restriction-mimetic mutations (*gpa2Δ, rpl6bΔ,* and *rpl31aΔ*) improve viability of the *orc2-1* mutation. (E) Model for how genetic and environmental modulation of rDNA origin firing affect replicative lifespan is presented by black lines with dashed lines denoting more speculative aspects of our model. Gray lines present links that are not addressed in this work.

Calorie restriction increases lifespan in organisms ranging from yeast to mammals. Coupled with the fact that these organisms all contain large numbers of rDNA repeats, and presumably replication origins, we speculated that calorie restriction might decrease origin firing at the rDNA, which in turn would alter global patterns of DNA replication in a manner that promotes longevity. Calorie restriction is known to affect rDNA through the reduction of ribosome biogenesis [42] and down-regulation of rRNA transcription [43], but its effects on rDNA replication have not been examined. To investigate the effect of calorie restriction on rDNA origin efficiency, we examined the rDNA replication intermediates in cells grown in 2% glucose (normal media) and 0.05% glucose (calorie-restricted media [20]). It is important to note that we are quantifying origin activity as the ratio of the bubble arc to the Y arc, which are both intermediates formed by DNA replication. These measurements therefore come from cells that are in S phase and changes to bubble∶Y ratios are not merely due to changes in number of S phase cells during nutrient deprivation. We found that BY cells with BY rDNA, grown under glucose restriction, had reduced rDNA origin activity by ∼60% when compared to the same cells grown in 2% glucose (Figure 7A). Calorie restriction had a more dramatic effect in BY cells with RM rDNA, where rDNA origin activity was reduced by >80% in 0.05% glucose. In fact, directly comparing the rDNA origin activity of the two strains in calorie-restricted medium, in which the promotion of origin activity via rRNA transcription is presumably reduced, underscores the intrinsic difference in rARS activity between the two strains.

Because the magnitude of the decrease in origin activity in response to glucose deprivation varied somewhat from experiment to experiment, we decided to assess the effect of dietary restriction on rDNA origin activity using an orthogonal approach, namely to examine rDNA replication in a panel of logarithmically growing, dietary restriction mimetic mutant strains including *gpa*2*Δ*, *gpr1Δ*, *hxk2Δ*, and *rpl6bΔ*. *GPA2* and *GPR1* encode signaling components of a G-protein coupled receptor, *HXK2* encodes a major hexokinase responsible for glucose phosphorylation during growth in glucose, and *RPL6B* encodes a component of the large ribosomal subunit. Loss of any of these genes acts as a calorie restriction mimetic, extending replicative lifespan through effects that are epistatic with calorie restriction [20], [44]. Compared to the wild type strain, the calorie restriction mimetic mutants that we analyzed all exhibited marked reduction in rDNA origin activity (∼45–60% reduction, Figure 7B), indicating that replication initiation at rDNA origins is influenced by nutrient signaling. Additionally, we found that effects of calorie restriction on rDNA origin activity are independent of both Sir2 and Fob1 (Figure 7C), as is the effect of calorie restriction on lifespan extension. The magnitude of inhibition of rDNA origin activity by calorie restriction mimetics suggests that calorie restriction may also profoundly improve genome-wide replication.

To investigate the possibility that calorie restriction extends life span by alleviating genome-wide replication stress, we examined *orc2-1* sensitivity in strains with *gpa*2*Δ*, *rpl6bΔ*, or *rpl31aΔ*, an additional calorie restriction mimetic mutant. Consistent with our observation that the maximum permissive temperature is raised for *orc2*-1 mutant in the presence of RM rDNA origins, deletion of each of the three genes also partially suppresses the temperature-sensitivity of *orc2-1* mutants (Figure 7D). Taken together, these results suggest that dietary restriction can promote genome-wide replication.

## Discussion

Our studies revealed that the rDNA locus is the key determinant of RLS in a cross between the laboratory BY4716 strain and the vineyard RM11-1a strain, with the vineyard rDNA locus providing robust lifespan extension. This modulation of lifespan by rDNA locus inheritance was found to be independent of Sir2 and Fob1, two previously established regulators of yeast longevity that interact with the rDNA locus [5], [11], suggesting that the rDNA locus itself has intrinsic properties important for lifespan modulation. Furthermore, lifespan extension by RM rDNA can occur without appreciable reduction in ERC accumulation, suggesting that modulation of ERC accumulation is not the primary mechanism by which RM rDNA regulates RLS. A polymorphism that reduces the activity of the RM rDNA origin of replication (rARS) is responsible for this lifespan extension and alters DNA replication not only at the rDNA but also on plasmids and other genomic origins. The rDNA locus was also implicated in a recent linkage study of RLS employing a laboratory strain and a clinical isolate as parents [45].

The RM strain has the rDNA allele that confers significant RLS extension, yet the lifespan of the RM strain itself is similar to that of the BY parental strain. These results suggest the presence of other loci in the RM parental strain that are detrimental for RLS relative to the BY alleles of those loci. The presence of such loci is demonstrated by the observation that the outcrossed BY strain with RM rDNA has a RLS of 38.6, which is 34% longer than the RLS of the RM parent at 28.8. Theoretically, this finding could be due to loci in the RM strain background that (1) interact with the RM rDNA such that the rDNA fails to confer a benefit; (2) decrease lifespan independently of the rDNA such that the RM parent has only an average lifespan despite receiving the benefits of the RM rDNA; or (3) a combination of rDNA-dependent and independent loci. While the backcrossing studies demonstrate that the RM rDNA allele extends RLS in both parental backgrounds, the RLS extension conferred by the RM allele is twice as large in the BY relative to the RM parental background (46% vs 23% increase), suggesting that average lifespan of the RM parent is likely due to a combination of additive and rDNA-interacting loci. Two observations suggest that the residual genetic variance that is not explained by the rDNA locus is controlled by numerous loci, each with a relatively small effect size: first, only minor loci registered in our genome-wide analysis (Table S2); and second, as mentioned earlier, the progeny from this cross showed striking transgressive segregation, with a continuous distribution of lifespans from much shorter to much longer than either parent, most likely indicating a large number of segregating loci with small and compensatory effects on RLS.

While long associated with yeast longevity, the exact role that rDNA plays in the aging process has remained elusive. Instability at the rDNA locus has been previously proposed as the rDNA's primary contribution [46], but the effect that a weakened rDNA origin may have on the integrity of the rDNA locus is complex. A weakened rDNA ARS would result in a greater inter-origin distance in the rDNA, reducing the number of stalled forks at the RFBs but increasing their persistence over S phase. It is therefore not obvious whether the opportunity for recombination would actually differ in strains with the different rDNA ARSs. Likewise, it is not clear how the maintenance of ERCs would be affected by the two different rDNA loci as the origin on an ERC will have the same ARS polymorphism as the resident rDNA locus with which it is competing for replication factors. Therefore, the maintenance of ERCs may not actually differ between the two strains. Our results confirm that there is no significant change in the frequency of RFB stalled forks (Figure S3) and that RM rDNA can extend RLS without appreciable reduction in ERCs. However, when amplified across the many repeats in the rDNA array, a weakened rDNA origin would significantly reduce the replication demand from the rDNA and alter replication elsewhere in the genome.

Although ours is the first report demonstrating that replicative lifespan is increased by weaker origin activity at the rDNA, it is not the first study to examine this question. Ganley *et al.* [31] showed that a mutation that reduces rARS activity slows down the amplification rate of rDNA and conversely, that the replacement of the wild type rARS with a stronger origin increases the amplification rate. Both manipulations, however, reduced RLS, albeit significantly more for the latter than for the former. Unlike our study, modification of rARS activity in a study by Ganley *et al.* involved insertion of 1.3 kb of DNA containing the *URA3* marker in the rDNA NTS. If one posits that this manipulation could compromise rDNA maintenance independently from any effects due to altered origin activation, the results from the two studies can be reconciled. Our models differ in the mechanisms through which events at the rDNA have implications for the genome as a whole. Specifically, Ganley *et al*. hypothesize that altered DNA replication triggers rDNA instability, which, in turn, decreases longevity by initiating a DNA damage response. In contrast, we hypothesize that decreased origin activity at the rDNA prolongs longevity by allowing reallocation of replication factors to the rest of the genome.

### rDNA and its influence on global DNA replication

We propose that a reduction in the number of actively replicating origins at the rDNA locus decreases competition for replication factors and therefore increases usage of non-ribosomal genomic origins, helping to ensure complete replication of the rest of the genome. rDNA origins represent a sizeable fraction of total cellular origins, viz., one third in the case of the laboratory strain. The RM rDNA, with both a weaker ARS and only 60% as many repeats relative to BY, could potentially liberate a significant amount of replication factors for use outside the rDNA. Consistent with this hypothesis, we found that strains with the less active RM rDNA origin sequence had increased activity at weak genomic and plasmid origins. We note, however, that our study examined the effect of RM rDNA on a very limited number of genomic and plasmid origins; assessment of the effect of weakened rDNA origins on activation of a full spectrum of plasmid origins as well as the global replication program will be required to fully evaluate our hypothesis. In further support of the idea that rDNA origins modulate the avilability of replication factors, we also found that the less active origins in the RM rDNA array also partially rescued lethality in *orc2-1* mutants. A similar rescue was reported by Ide *et al*. [34] when they characterized clones that were able to suppress the *orc1-4* and *orc2-1* temperature-sensitive phenotype. A majority of the rescued strains possessed a dramatically smaller chromosome XII, a result of severe reductions to the rDNA array. This flexibility in rDNA size suggests that it is a dynamic structure that can respond to and compensate for replication constraints.

### How does DNA replication affect lifespan?

Genome-wide, origins differ in their efficiency and timing of firing. Some origins are very efficient, firing in nearly all cells in the population. Other origins, perhaps less efficient at recruiting all of the necessary components for origin firing, will be passively replicated by forks from adjacent origins in a portion of cells in the population. The stochastic nature of these less-efficient origins can give rise to situations in which adjacent active origins are far enough apart that the converging replication forks will not meet by the time the cell initiates mitosis. Such a “random replication gap” problem [47] would presumably lead to a cell cycle delay or to chromosome and/or nuclear segregation defects. Replication initiation also varies temporally between different genomic origins, with late firing origins important for late S-phase synthesis of remaining stretches of unreplicated DNA. Since rDNA is a later replicating region of the genome (Alvino, unpublished) and most likely consists of later-firing origins, having fewer active origins at the rDNA array would allow reallocation of replication resources during a critical period in late S-phase when unreplicated genomic gaps need to be resolved. We hypothesize that this replication problem becomes more acute with age and that a weaker origin in the rDNA repeats can be enough to stave off the random gap problem to allow cells to complete a greater number of cell divisions.

This hypothesis raises the question of whether the RM rDNA is relieving replication stress outside of the rDNA at the expense of increasing stress within the rDNA. Due to its repetitive and hyper-recombinogenic nature, however, the rDNA may have options for repair of unreplicated regions that are unavailable for the rest of the genome -for example, single stranded annealing and homologous recombination between direct repeats [48]. Therefore rDNA may be less vulnerable to crises resulting from random replication gap induced double strand breaks.

### Could reduced rDNA origin efficiency contribute to lifespan extension from dietary restriction?

We are proposing that the difference in the genetic potential for origin firing in the laboratory and vineyard strains elucidates a fundamental mechanism by which cells sense and respond to dietary restriction. Specifically, we suggest that nutrient deprivation causes decreased rRNA transcription, resulting in increased nucleosome occupancy at the rDNA, which in turn would reduce firing of rDNA origins of replication. In support of this idea, we found that dietary restriction dramatically reduces rDNA origin firing. Furthermore, it is well established that in situations in which growth is reduced, such as with calorie restriction or TOR pathway inhibition, there is a dramatic down-regulation of genes involved in ribosome biogenesis [49]. rRNA transcription is particularly attuned to growth conditions and the number of repeats transcribed decreases when cells are shifted from nutrient-rich to nutrient-poor media [43]. During such a dietary shift, the rDNA locus undergoes a dramatic increase in nucleosome occupancy, and the rDNA origin itself becomes populated by nucleosomes [50]. Since nucleosome occupancy and local nucleosome positioning exert potent effects on origin selection and function [33], we propose that rRNA transcription-induced nucleosome alterations couple dietary restriction with regulation of rDNA origin firing.

In metazoans, in which DNA replication origins are not specified by DNA sequence and ORC proteins do not exhibit sequence specificity in vitro, chromatin features and transcription have particularly important roles in shaping DNA replication initiation [33] [51]. In human rDNA, transcriptionally active and transcriptionally silent repeats differ in their timing, location, and pattern of DNA replication initiation [52], suggesting that growth-related stimuli, similarly to what we observed in yeast upon dietary restriction, could alter rDNA replication dynamics with possible consequences for genome-wide replication. In further support of this idea, genome wide trans-effects of the rDNA locus have been observed in *Drosophila melanogaster* at the transcriptional level [53], raising the possibility that the rDNA locus may also affect global DNA replication initiation patterns. Highly conserved processes such as the rRNA transcriptional response to nutrient deprivation and resulting alterations in DNA replication dynamics present an appealing mechanism to contribute to the longevity-promoting effects of dietary restriction across a wide range of species [6].

By focusing on rDNA origin activation and its impact on global rDNA replication, our model accounts for a large body of experimental observations. For example, Sir2 is not only a central determinant of RLS but also a potent suppressor of origin activation at the rDNA [54]. It is thus possible that suppression of rARS activation is the mechanism through which Sir2 promotes longevity. Likewise, deleting *FOB1* inactivates the unidirectional replication fork barrier and permits replication from rDNA origins to be bidirectional. Replication of the rDNA locus would then require fewer active origins (Figure 7C). We also note that our model can also accommodate a role of ERCs in shortening the lifespan of old mothers since excessive ERCs would also titrate replication factors away from the rest of the genome.

In summary we suggest that down-regulation of replication initiation in the rDNA plays a central role in life span extension (Figure 7E). The importance of keeping initiation potential low in the rDNA is suggested by the inherently weak nature of the rDNA ARS [27] and is further supported by the specific rARS polymorphism that we found in RM and other wild strains. We furthermore suggest the beneficial effects of calorie restriction act through the rDNA replication initiation pathway (Figure 7E): (1) calorie restriction decreases rDNA transcription via nutrient-sensing pathways; (2) decreased transcription causes decreased firing of rDNA origins; (3) decreased firing of rDNA origins frees up significant replication factors for the rest of the genome; and (4) these increased replication factors alleviate the random replication gap problem, a lethal problem that becomes worse with age.

## Materials and Methods

### Yeast strains and media

Yeast strains, plasmids, and oligos used are listed in Table S1. Yeast experiments were carried out using standard YPD medium (2% glucose, 1% yeast extract, 2% peptone) unless otherwise noted (e.g., calorie restriction). The segregant library has been previously described [55]. Gene deletion mutants were either from yeast ORF deletion collection or were created using standard PCR transformation methods. The Saccharomyces Genome Resequencing Project (SGRP) strains were provided by the National Collection of Yeast Cultures (NCYC) [30], [56]. Strains with reduced rDNA copy numbers were generated as described previously [35] using pRDN-hyg1 provided by the Liebman Lab [57]. Briefly, the plasmid pRDN-hyg1, which contains a single rDNA repeat with a recessive hygromycin resistance mutation in the 18S rRNA sequence, was introduced into strains containing native number of rDNA repeats. The transformants were subjected to hygromycin resistance selection, which selects for mutants that have lost most of the copies of chromosomal rDNA repeats. The size of rDNA in the resulting strains was measured using CHEF gel electrophoresis and quantitative Southern blotting. JRY7459, the S288c strain containing *orc2-1* mutation in the S288c background, was obtained from the Rine lab [41] and crossed with strains of interest to generate tetrads for temperature sensitivity assays. Backcrossed rDNA strains in the BY background were generated by crossing an rDNA-tagged BY parent strain, in which HYG (HpHMX4) was inserted in the rDNA array, to the RM parent strain. We selected multiple spores without resistance to hygromycin B (have RM rDNA instead of the tagged BY rDNA), which were then again crossed with the rDNA-tagged BY parent strain. The rDNA of each strain was sequenced after 8 backcrosses to verify that the desired rDNA sequence was maintained.

### Replicative lifespan analysis

Replicative lifespan studies were conducted as previously described by Kaeberlein *et al.* [58]. All lifespan studies were conducted on YPD plates (2% glucose) and 40 individual cells (virgin mothers) were analyzed for each strain, except for the segregant strain studies in which 20 cells were analyzed. Statistical significance between strain lifespans was determined using Mantel-Haenszel logrank test.

### QTL mapping

Genome-wide linkage analysis of segregant data was performed using the publicly available R/qtl software. Effects of RM/BY rDNA locus inheritance were examined using R (box plots) and Excel (student's t-test).

### ERC analysis for young and old mother cells

Old cells were harvested using the biotinylation/streptavidin purification technique described previously [10], [59]: 10^8^ cells from logarithmically growing cultures were biotinylated using EZ-Link sulfo-NHS-LC-biotin (Pierce), cultured in YPD for 10 doublings, labeled with streptavidin magnetic beads (Miltenyi Biotec), and purified using an autoMACs Pro Separator (Miltenyi Biotec). Purification of old mother cells was confirmed by counting bud scars using calcoflour staining. Purified old mother cells exhibited a median of 8 bud scars. Young cells were harvested from logarithmically growing YPD cultures. For ERC Southern blot analysis, 1 µg of undigested genomic DNA was resolved by gel electrophoresis (0.8% agarose, 1 V/cm for 36 hours) and blotted to a nylon membrane. Membranes were then hybridized with a ^32^P-labeled rDNA probe, generated from an rDNA NTS2 template PCR-amplified from genomic DNA using primers 5pNTS2_XhoI/3pNTS2_BamHI (Table S1), visualized using a Personal Molecular Imager (Bio-Rad), and quantified using Quantity One software.

### Analysis of rDNA size by quantitative Southern blot and CHEF gel

For rDNA copy number quantification by Southern blot, genomic DNA was harvested from saturated 3 mL cultures, digested overnight with BglII, resolved by gel electrophoresis, and blotted to a nylon membrane. Abundance of rDNA and single-copy *MCM2* was visualized using ^32^P-labeled probes from rDNA NTS2 and MCM2 templates amplified from genomic DNA, and quantified using ImageJ. Relative rDNA copy number was determined by normalizing rDNA band intensity to the intensity of single-copy *MCM2* [60]. Chromosome XII size was resolved by CHEF (clamped homogeneous electric field) gel electrophoresis as previously described by Ganley *et al.* [31]. For each strain, genomic DNA from saturated cultures from 3 individual colonies was harvested in 1% agarose plugs [61], each plug containing approximately 10^8^ cells. Chromosomes were visualized by ethidium bromide staining and/or Southern blotting.

### rDNA sequence analysis

The S288c (BY) rDNA sequence was obtained from the Saccharomyces Genome Database [62], [63] and the RM11-1a rDNA sequence was obtained from the Broad Institute *Saccharomyces cerevisiae* RM11-1a Sequencing Project [64]. To verify polymorphisms found in RM and BY rDNA, the 2.2 kb NTS sequence (NTS1/5S/NTS2) was amplified from genomic DNA using primers 5pNTS1_XhoI/3pNTS2_BamHI (Table S1), cloned into pRS416, and sequenced from multiple clones. Genomic DNA from BY4716 and RM11-1a parental strains and the backcrossed “BY strain with RM rDNA” was further analyzed using Illumina sequencing. rDNA repeat heterogeneity was estimated using read abundance from each. rDNA sequences from the SGRP collection [56] were examined with BLAST searches performed on the SGRP database [29].

### Assessment of ARS efficiency via plasmid transformation assay

*S. cerevisiae* origin sequences were PCR-amplified from genomic DNA and cloned into the ARS-free pU18-KanMX-LwCEN vector previously used by Di Rienzi *et al.* [36]. 50 ng of each plasmid was transformed into 10^8^ cells of logarithmically growing culture. Transformation cultures were allowed to recover 24 hours on YPD plates before being replica-plated onto selective YPD+G418 media. Colony formation was visualized after 48 hours at 30°C, 72 hours for plasmids with RM rARS or *L. waltii* ARSVII-929.

### 2D gel electrophoresis

To examine replication intermediates, two dimensional (2D) gel electrophoresis was performed as described previously [65]. Genomic DNA from logarithmically growing cultures was harvested in 0.5% agarose plugs or by modified version of the method of Huberman [66] and digested with NheI (rDNA and *ARS14*), StyI (*ARS1*), or HincII/NcoI (*ARS605*). Replication intermediates were visualized on film and analyzed using a Personal Molecular Imager System (Bio-Rad) and Quantity One software. Relative origin activity was estimated by normalizing the intensity of bubble-arc (active origin) intermediates to the Y arc (passive replication).

## Supporting Information

Figure S1 RM rDNA does not alter cell growth rate or cell size. (A) Growth curve of BY and BY w/RM rDNA in YPD or YP+0.05% glucose. (B) Coulter counter data indicating overlap of cell size distribution of logarithmically growing BY and BY w/RM rDNA strains.
(TIF)

Figure S2 Maintenance of a plasmid with a weak origin is influenced by the rDNA origin sequence, related to Figure 5. High-frequency transformation assay of plasmids with different ARSs was done as described for Figure 5B. Host strains that have rDNA loci with the RM-like rARS (RM, BY w/DBVPG6765 rDNA) replicate weak-origin plasmids better than host strains with the BY-like rARS (RM w/BY rDNA, BY, BY w/Y12 rDNA).
(TIF)

Figure S3 Strains with BY rDNA and RM rDNA have the same frequency of stalled forks at the RFB. (A) Diagram illustrating the position of stalled replication forks at the RFB from NheI-digested rDNA fragments run on a 2D gel. To estimate differences in relative frequencies of RFB-stalled forks between strains, quantified replication intermediates at the RFB spot were compared to intermediates from the ascending Y arc (gray dashed region). (B) The 4.7 kb NheI rDNA fragments from a BY strain with intact *FOB1* and unaltered copy numbers of either BY rDNA (150 repeats) or RM rDNA (90 repeats) are examined by Southern blotting of a 2D gel. The ratio of RFB∶Y was defined as 1.00 for the BY rDNA strain and 0.96 for the RM rDNA strain.
(TIF)

Table S1 Yeast strains, plasmids, and primers used.
(XLS)

Table S2 Loci linked to the control of replicative lifespan in the RM/BY segregant library (see Figure 1).
(XLS)
