# Supplementary figures and images for: A Natural Polymorphism in rDNA Replication Origins Links Origin Activation with Calorie Restriction and Lifespan
Source: PLoS Genet. 2013 Mar 7;9(3):e1003329. doi: 10.1371/journal.pgen.1003329 (PMC3591295; doi:10.1371/journal.pgen.1003329)

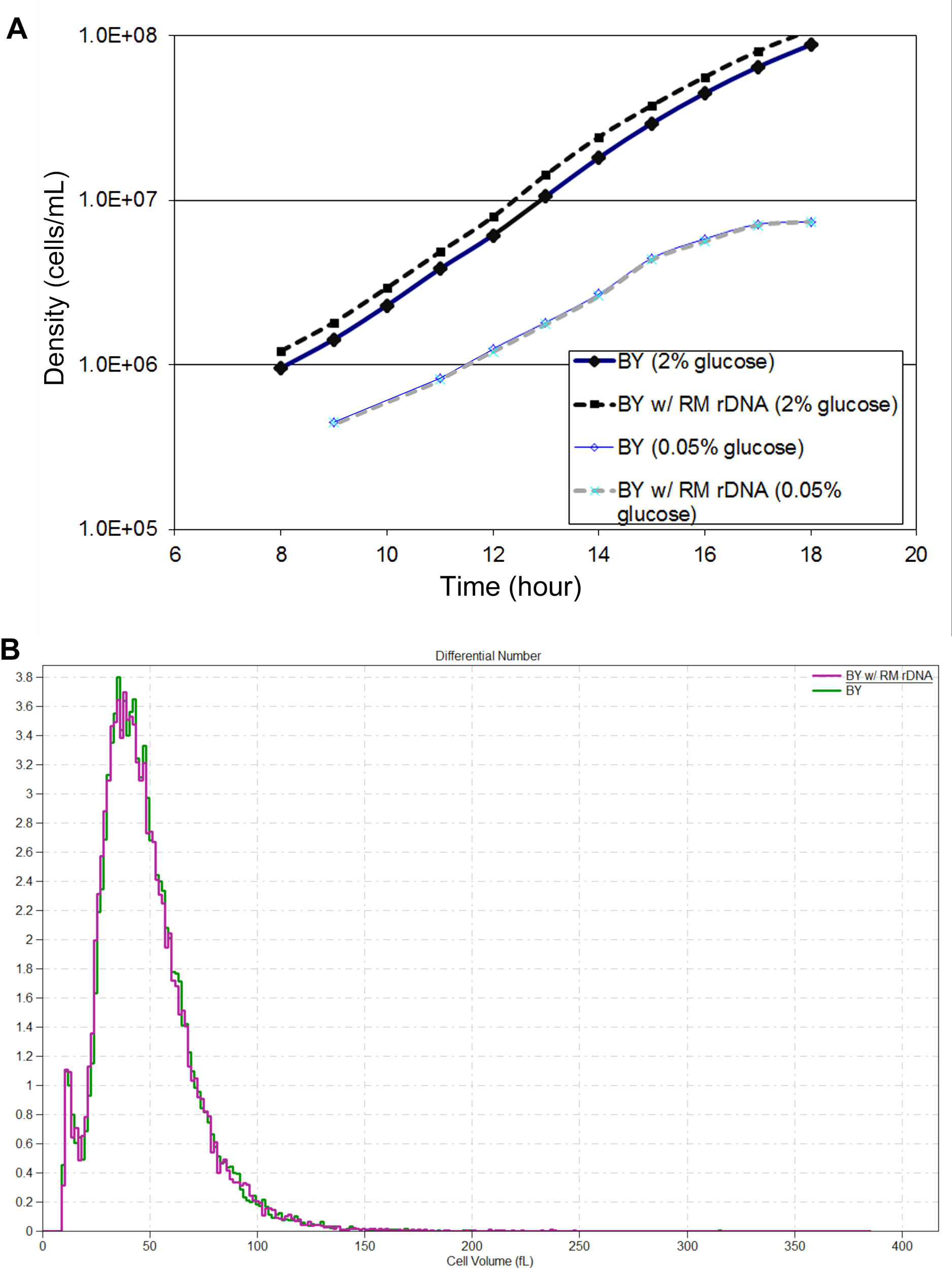

Supplement: Figure S1 — RM rDNA does not alter cell growth rate or cell size. (A) Growth curve of BY and BY w/RM rDNA in YPD or YP+0.05% glucose. (B) Coulter counter data indicating overlap of cell size distribution of logarithmically growing BY and BY w/RM rDNA strains. (TIF) [file pgen.1003329.s001.tif]

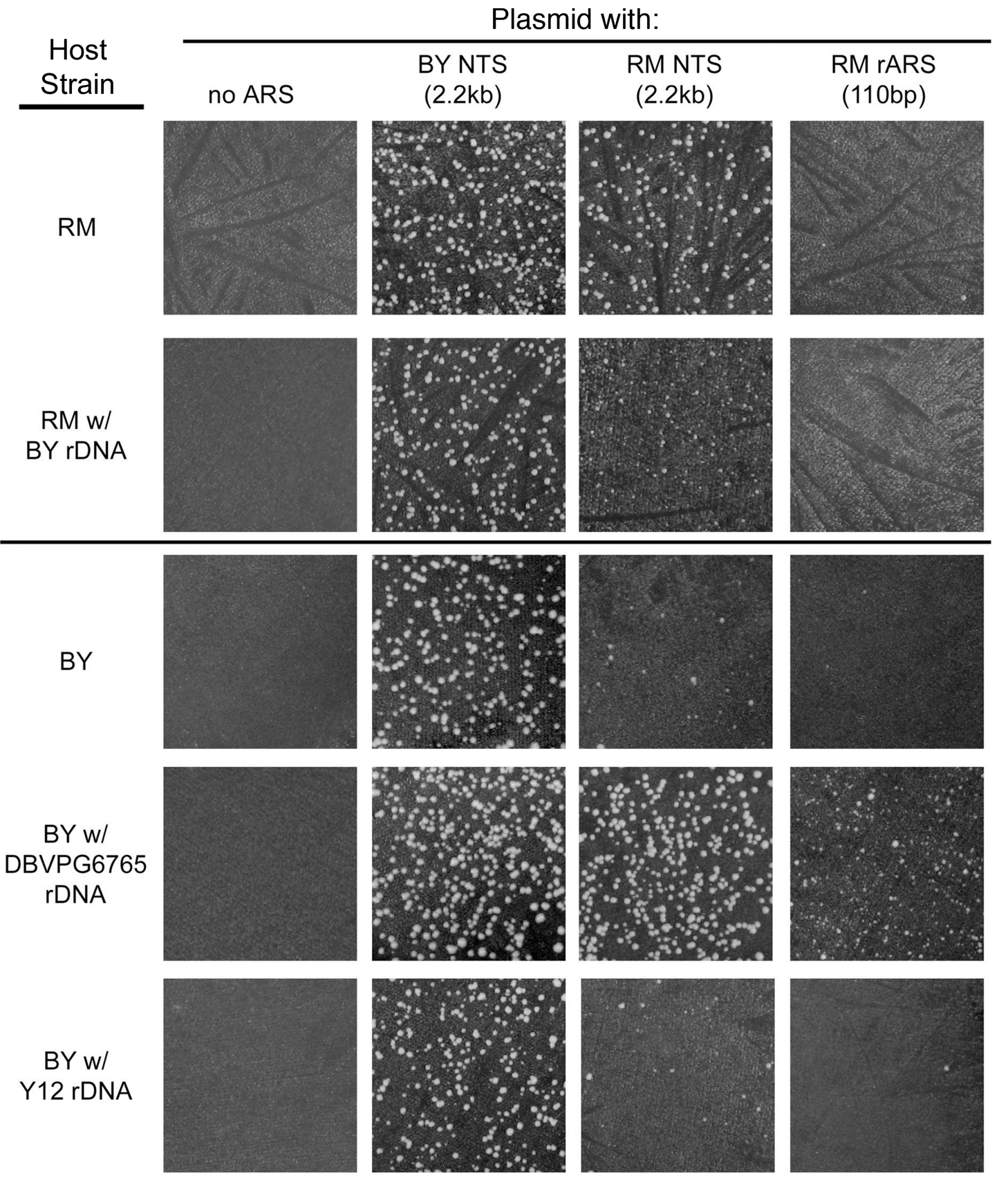

Supplement: Figure S2 — Maintenance of a plasmid with a weak origin is influenced by the rDNA origin sequence, related to Figure 5. High-frequency transformation assay of plasmids with different ARSs was done as described for Figure 5B. Host strains that have rDNA loci with the RM-like rARS (RM, BY w/DBVPG6765 rDNA) replicate weak-origin plasmids better than host strains with the BY-like rARS (RM w/BY rDNA, BY, BY w/Y12 rDNA). (TIF) [file pgen.1003329.s002.tif]

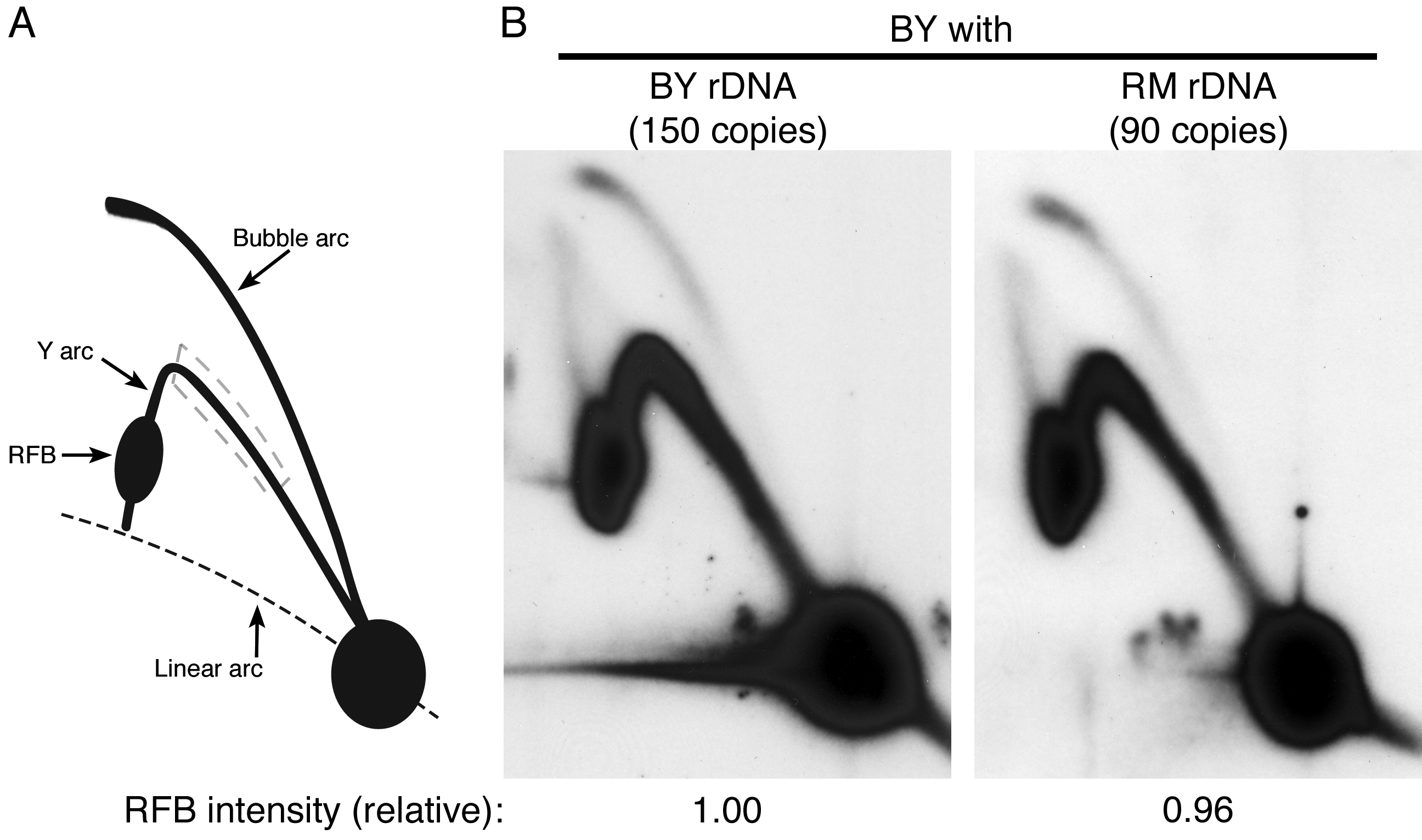

Supplement: Figure S3 — Strains with BY rDNA and RM rDNA have the same frequency of stalled forks at the RFB. (A) Diagram illustrating the position of stalled replication forks at the RFB from NheI-digested rDNA fragments run on a 2D gel. To estimate differences in relative frequencies of RFB-stalled forks between strains, quantified replication intermediates at the RFB spot were compared to intermediates from the ascending Y arc (gray dashed region). (B) The 4.7 kb NheI rDNA fragments from a BY strain with intact FOB1 and unaltered copy numbers of either BY rDNA (150 repeats) or RM rDNA (90 repeats) are examined by Southern blotting of a 2D gel. The ratio of RFB∶Y was defined as 1.00 for the BY rDNA strain and 0.96 for the RM rDNA strain. (TIF) [file pgen.1003329.s003.tif]
